# Organization and evolution of the UK far-right network on Telegram

**DOI:** 10.1007/s41109-022-00513-8

**Published:** 2022-11-15

**Authors:** Alexandre Bovet, Peter Grindrod

**Affiliations:** 1 Department of Mathematics and Digital Society Initiative, University of Zurich, Zurich, Switzerland; 2 Mathematical Institute, University of Oxford, Oxford, UK

**Keywords:** Social network, Communication, Social media, Community detection, Directed network, Extremism, Organization, Evolution

## Abstract

The instant messaging platform Telegram has become popular among the far-right movements in the US and UK in recent years. These groups use public Telegram channels and group chats to disseminate hate speech, disinformation, and conspiracy theories. Recent works revealed that the far-right Telegram network structure is decentralized and formed of several communities divided mostly along ideological and national lines. Here, we investigated the UK far-right network on Telegram and are interested in understanding the different roles of different channels and their influence relations. We apply a community detection method, based on the clustering of a flow of random walkers, that allows us to uncover the organization of the Telegram network in communities with different roles. We find three types of communities: (1) upstream communities contain mostly group chats that comment on content from channels in the rest of the network; (2) core communities contain broadcast channels tightly connected to each other and can be seen as forming echo chambers; (3) downstream communities contain popular channels that are highly referenced by other channels. We find that the network is composed of two main sub-networks: one containing mainly channels related to the English-speaking far-right movements and one with channels in Russian. We analyze the dynamics of the different communities and the most shared external links in the different types of communities over a period going from 2015 to 2020. We find that different types of communities have different dynamics and share links to different types of websites. We finish by discussing several directions for further work.

## Introduction

Telegram is a free and open-source instant messaging software that was launched in 2013 by the two Russian brothers Nikolai and Pavel Durov. Its usage has steadily increased over the years to a reported number of active monthly users of 400 million in April 2020^1^. Telegram offers end-to-end encryption of messages, voice and video calls and of “secret” chats. It also allows its users to create and use freely accessible public group chats and broadcasting channels. The platform is popular in fringe groups and for illegal activities that often take refuge on Telegram after having been banned on less permissive platforms such as Twitter or Facebook (Rogers 2020). For example, until a joined operation by Europol and Telegram in November 2019 that removed 43,000 terrorist-related bots and channels, the terrorist group Islamic State of Iraq and the Levant (ISIS) recommended Telegram to its supporters and members and used it to promote radicalism and to coordinate operations (Prucha 2016)^2^. In particular, Telegram gained popularity among the far-right movements in the US, UK and Europe (Urman and Katz 2022; Walther and McCoy 2021). These groups make use of public Telegram channels and group chats to disseminate hate speech, disinformation and conspiracy theories (Walther and McCoy 2021).

Several works have investigated Telegram in relation to the effect of de-platforming of extreme Internet celebrities (Rogers 2020), the growth of channels supporting hateful ideologies (Walther and McCoy 2021), the dissemination of misinformation (Knuutila et al. 2020) or the toxicity of the language used in the groups/channels related to the far-right conspiracy theory of QAnon (Hoseini et al. 2021). However, little work has been done to understand the organization of the far-right network of channels and group chats. Urman and Katz (2022) investigated the structure and evolution of the network of far-right channels using standard community detection (Modularity optimization) and found communities divided mostly along the ideological and national lines, with the communities related to 4chan imageboard and Donald Trump’s supporters being the most influential. Their analysis of the network's evolution suggests that the start of its explosive growth is due to the mass bans of far-right actors on mainstream social media platforms.

Here, we use the novel community detection method *flow stability* for temporal networks based on the clustering of a flow of random walkers diffusing along its edges (Bovet et al. 2022) adapted to static directed network. This framework allows us to uncover the organization and the influence relations of the different Telegram communities. Compared to classical community detection (Urman and Katz 2022), our approach not only reveals tightly connected clusters but also groups together channels that play similar roles in the system. Channels/groups that influence the same set of channels (i.e. that have similar outgoing flows) or that are influenced by the same set of channels/groups (i.e. that have similar ingoing flows) are clustered together. In this way, we can classify communities in a directed core-periphery model as either: periphery communities (upstream or downstream) or core communities. Compared to standard directed core-periphery detection methods (Elliott et al. 2020), our method can find several communities in the periphery or in the core. Upstream communities contain channels that mostly have outgoing links (pointing to the same channels), i.e. that are mostly commentators of channels from other communities. Channels/groups inside core communities have strong links with each other, actively participate in discussions across channels, and can be seen as forming echo chambers. Downstream communities contain popular channels/groups that are highly referenced by other similar channels.

We identify the most important channels in the main communities and the domain names of the most shared external links. We also investigate the evolution and dynamics of the different communities. These analyses reveal that upstream, core and downstream communities play different roles in the system as they contain different types of channels, link to different types of external websites and have different dynamics. We find that channels and group chats related to the far-right are grouped into three main communities: an upstream community with the main group chats that, a core community with the main broadcast channels of far-right groups and activists and a downstream community with the main channels influencing discussions in the two other communities. We also find a parallel sub-network of channels that are mostly in the Russian language. We finish by discussing several directions for further work.

## Results

### Dataset and network reconstruction

To build our dataset, we first used a large open dataset of Telegram messages containing over 27.8K channels and 317M messages from 2.2M unique users (Baumgartner et al. 2020). We refer to this dataset as the Pushshift dataset. This dataset covers messages from September 2015 until November 2019 and was collected using a snowball sampling approach starting from a seed list of 250 primarily English-language public broadcast channels and public group chats, among which 124 channels focus on right-wing extremist politics. All the available messages from each of those channels were collected and new channels are discovered each time content forwarded from a channel that is not already in the dataset is found. The messages from the new channels were then collected and the procedure was repeated using the forwarded content of the newly discovered channels (Baumgartner et al. 2020). To find messages relevant to the UK far-right, we selected all Telegram messages from the Pushshift dataset containing the term **britainfirst** in their content. This includes messages containing links to the Britain First Telegram channel and URLs using the term in their address, such as URLs pointing to Britain First’s website. We choose to focus on Britain First because it is a central organization in the UK far-right movement^3^ and because a manual inspection of the Pushshift dataset revealed that the official Britain First Telegram channel was a prominent channel for keywords related to the far-right in the UK and that other active British far-right organizations (e.g., British National Party, National Front or Sonnenkrieg Division^4^) did not have an active presence in the Pushshift dataset. Among all the URLs in the messages we found, 98.6% are URLs linking to the Britain First channel or to other Telegram channels (in the form t.me/channel_name). These come from messages containing a list of links pointing to several related channels that are frequently observed in Telegram. The other URLs are linking to secret Telegram chats (0.8%) and external websites (0.6%). This results in a seed set of 2729 posts from April 6th, 2019 to October 14th, 2019 from 258 channels/groups. We then expanded our dataset by collecting (using the Telegram API) all existing messages from all the public channels/groups that appeared in our initial seed set, i.e. all the messages of the 258 channels from the Pushshift dataset and additional channels we found through forwarded content, links to join them or mentions. As Telegram users are free to delete their messages, we were only able to collect messages that had not been deleted. The resulting dataset contains 7 million messages from 12,564 channels posted between September 2015 and January 2021.

**Fig. 1:**
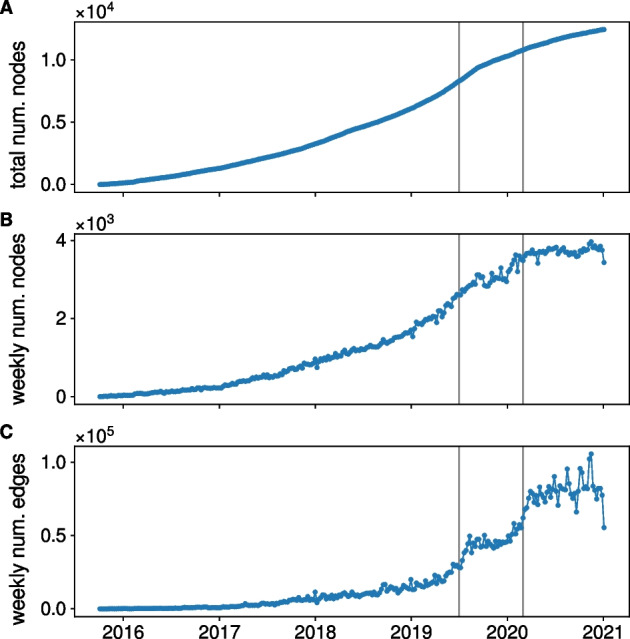
Evolution of the network of Telegram public channels/groups related to the far-right movement. We show the cumulative number of nodes in the network (**A**) and the weekly number of unique nodes (**B**) and edges (**C**) in the network. The gray vertical lines indicate July 1st, 2021 and March 1st 2022 corresponding to the separation in three datasets we use

We build a directed weighted network where nodes represent channels/groups and edges represent the potential flow of users between channels: an edge from node A to node B can represent a mention of node B in node A, a URL link pointing to node B posted in node A or message from node B forwarded in node A. With this convention, relations of potential influence between channels follow the opposite direction of edges. Figure 1 shows the evolution of the network size and of the weekly number of edges and unique nodes.

Figure 1C shows the number of edges, counting multi-edges between the same two nodes. It is therefore a measure of the weekly activity between channels in the network. We observe two significant rapid increases in the number of weekly edges: the first in mid-2019 and the second in early 2020, corresponding to the start of the COVID-19 pandemic. The first increase in 2019 has also been observed by Urman and Katz (2022). They identified it as being connected to British far-right actors joining Telegram after being banned from Facebook and Instagram at that time. We use these two instants to separate our dataset into three networks. The first network spans the interval from September 2015 until June 30th, 2019 and corresponds to the early phase of the network that see a constant and slow increase in the number of channels and activity. The second one spans the interval from July 1st, 2019 until February 29th, 2020 and corresponds to a time of marked increase in activity in the network. The last one covers the first year of the COVID-19 pandemic and corresponds to an additional increase in activity. It starts on March 1st, 2020 and finished at the end of the collection period, December 31st, 2020.

Table 1 shows the number of nodes and edges of the three networks as well as the number of mentions, forwards and links in each network. In each network, forwarding is the dominating type of activity between channels. For the remainder of the analysis, we represent the three networks as weighted directed networks where the weight of each edge is given by the total number of interactions, in the same direction, between two channels.

**Table 1:** Statistics of the telegram networks corresponding to the three periods we consider

	Period	Mention	Forward	Link	Num. nodes	Num. single edges
G1	Sep. 2015 to June 2019	325,774	820,516	175,772	8294	123,161
G2	July 2019 to Feb. 2020	371,539	938,927	290,252	7942	191,209
G3	March 2020 to Dec. 2020	772,468	1,981,304	790,842	8570	377,397

We report the numbers for the three types of interactions between channels: mention of another channel, forwarding a message from another channel and posting a link to another channel. We also report the number of single edges, i.e. the number of directed edges between channels discarding parallel edges between the two same channels

### Network clustering

In order to understand the organization of the telegram channels we employ a clustering method that allows us to take into account the direction of the edges in the networks. As edges represent the potential flow of users in this network, this approach allows us to reveal the asymmetrical relations between clusters, or communities, in terms of influence and flow of users. We use the flow stability method (Bovet et al. 2022) that clusters nodes together based on the similarity of a diffusive flow starting on them. This method generalizes the Markov stability (Delvenne et al. 2010; Lambiotte et al. 2014) method to non-stationary dynamics and is therefore well suited to directed networks that are not strongly connected, i.e. where a diffusive flow does not reach a non-trivial stationary state. The clustering yields two partitions obtained by clustering the covariance matrix of the diffusive process in forward and backward time. Here, we adapt the method for static directed networks by considering two transition matrices for two continuous time random walk processes: one following the edges (forward) and one going in the reverse direction of the edges (backward). The two transition matrices are given by $\textbf{T}(t)_\text {f}=e^{-t \textbf{L}_\text {f}}$ and $\textbf{T}(t)_\text {b}=e^{-t \textbf{L}_\text {b}}$, respectively. The forward and backward Laplacians are given by $\textbf{L}_\text {f}=\textbf{I}-\textbf{D}_\text {out}^{-1}\textbf{A}_\text {f}$ and $\textbf{L}_\text {b}=\textbf{I}-\textbf{D}_\text {in}^{-1}\textbf{A}_\text {b}^T$, where $\textbf{A}_\text {f}$ is the forward adjacency matrix of size $N_\text {f}\times N_\text {f}$, obtained by iteratively removing nodes that have an out-degree equal to zero, $\textbf{A}_\text {b}$ is the backward adjacency matrix of size $N_\text {b}\times N_\text {b}$, obtained by iteratively removing nodes that have an in-degree equal to zero, $\textbf{D}_\text {out}=\text {diag}(\textbf{A}_\text {f}\textbf{1})$ and $\textbf{D}_\text {in}=\text {diag}(\textbf{A}_\text {b}^T\textbf{1})$. Using uniform initial conditions, $\textbf{p}_\text {f}(0)=\frac{1}{N_\text {f}}{\textbf{1}}$ and $\textbf{p}_\text {b}(0)=\frac{1}{N_\text {b}}{\textbf{1}}$, the covariance matrices of both processes are given by (Bovet et al. 2022)

1 $$\begin{aligned} \textbf{S}_\text {forw}(t)&=\textbf{P}_\text {f}(0)\textbf{T}_\text {f}(t)\textbf{T}_\text {f}^\text {inv}(t) - \textbf{p}_\text {f}(0)^T\textbf{p}_\text {f}(0) \end{aligned}$$

2 $$\begin{aligned}&=\frac{1}{N_\text {f}}\textbf{T}_\text {f}(t)\textbf{T}_\text {f}^\text {inv}(t) - \frac{1}{N^2_\text {f}} \overleftrightarrow {{\textbf{1}}} \end{aligned}$$

and

3 $$\begin{aligned} \textbf{S}_\text {back}(t)&=\textbf{P}_\text {b}(0)\textbf{T}_\text {b}(t)\textbf{T}_\text {b}^\text {inv}(t) - \textbf{p}_\text {b}(0)^T\textbf{p}_\text {b}(0) \end{aligned}$$

4 $$\begin{aligned}&=\frac{1}{N_\text {b}}\textbf{T}_\text {b}(t)\textbf{T}_\text {b}^\text {inv}(b) - \frac{1}{N^2_\text {b}} \overleftrightarrow {{\textbf{1}}} \end{aligned}$$

where $\overleftrightarrow {{\textbf{1}}}$ is the all-ones matrix, $\textbf{T}_\text {f}^\text {inv}(t)$ and $\textbf{T}_\text {b}^\text {inv}(t)$ are the transition matrices of the inverse processes, i.e. satisfying $\textbf{p}_\text {f}(t)\textbf{T}_\text {f}^\text {inv}(t)=\textbf{p}_\text {f}(0)$ and $\textbf{p}_\text {b}(t)\textbf{T}_\text {b}^\text {inv}(t)=\textbf{p}_\text {b}(0)$ respectively. They are given by Bayes’ Theorem as $\textbf{T}_\text {f}^\text {inv}(t)=\textbf{P}(t)^{-1}\textbf{T}_\text {f}^T(t)\textbf{P}(0)$ and $\textbf{T}_\text {f}^\text {inv}(t)=\textbf{P}(t)^{-1}\textbf{T}_\text {f}^T(t)\textbf{P}(0)$. The element (*i*, *j*) of the covariances encodes the probability for two random walkers starting on nodes *i* and *j* to be on the same node at time *t* minus the same probability for two independent random walkers. The two partitions found by clustering the two covariance matrices in diagonal blocks, therefore, group together nodes in communities of nodes that act as similar “sources”, for the forward covariance, or “sinks” for the backward covariance, of the flow. The forward and backward quality functions are given by the traces of the clustered forward and backward covariance matrices (Delvenne et al. 2010) and allow one to find the best forward and backward partitions with a range of optimization algorithms such as the Louvain algorithm. As in the Markov stability framework (Delvenne et al. 2010), the time *t* plays the role of a resolution parameter. As time increases, the random walk covers larger distances in the network and larger communities are found.

We then combine the forward and backward partitions in one partition. Nodes that are only in $\textbf{A}_\text {f}$ are clustered according to their forward communities, nodes that are only in $\textbf{A}_\text {b}$ are clustered according to their backward communities and nodes that are in both networks are clustered according to the intersection of the forward and backward partitions. We note that some nodes of the original network may have been removed in both $\textbf{A}_\text {f}$ and $\textbf{A}_\text {b}$ and therefore absent from the forward and backward partitions. In practice, they represent a very small number of nodes. We consider an additional community containing those nodes that have, by construction, little importance in terms of source or sinks in the network.

**Fig. 2:**
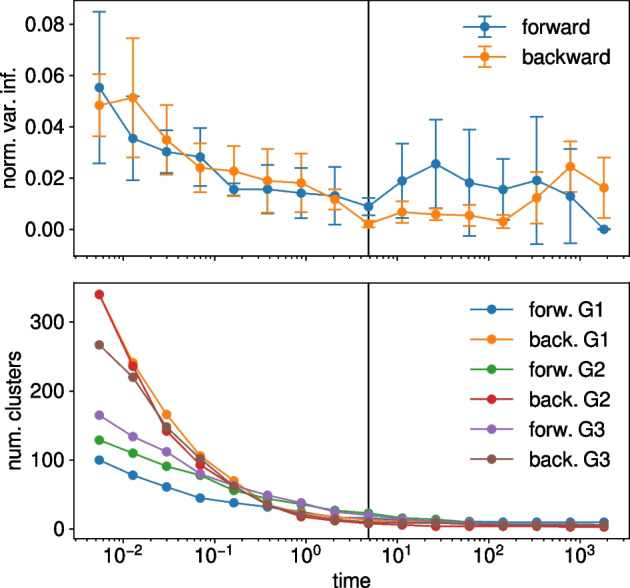
Scan in resolution of the flow stability method. Normalized variation of information of the results as a function of the duration of the diffusive process (top). The average and standard deviation computed across the three networks are shown. Number of clusters in the best forward and backward partitions as a function of the time (bottom). The time plays the role of a resolution parameter

We vary the time parameter and perform the optimization 50 times at each time point with the Louvain algorithm. The average normalized variation of information (NVI) of the ensemble of 50 partitions found at each time measures the stability of the solutions and allows one to find natural scales of the system corresponding to minima of the NVI (Lambiotte et al. 2014). Figure 2 shows the average and standard deviation of the NVI taken across the three networks. We choose the value $t=4.8$ as our resolution as it corresponds to a minimum for the forward and backward NVI (indicated by a vertical line in Fig. 2).

**Fig. 3:**
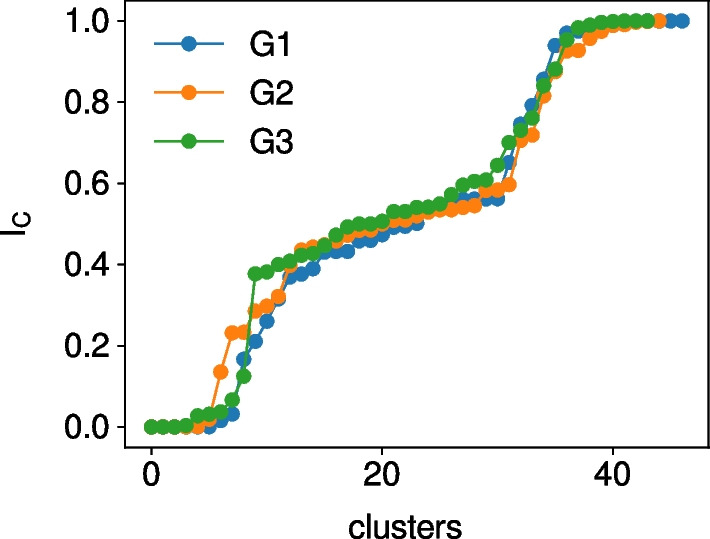
Average proportion of incoming edges per clusters

In order to classify clusters as being upstream, downstream or core clusters we characterize how many nodes in each cluster tend to rather have incoming or outgoing edges by measuring the average proportion of incoming edges of each cluster. For a cluster *C* this gives $I_C = \frac{1}{|C|}\sum _{i \in C} \frac{s_i^\text {in}}{s_i^\text {in} + s_i^\text {out}}$, where $s_i^\text {in}$ and $s_i^\text {out}$ are the in- and out-strengths of node *i*, respectively. Figure 3 shows the sorted values of $I_C$ for the three networks. Three plateaus of low, middle, and high values are visible. We choose to classify clusters with $I_C$ values higher than 0.8 as *downstream*, the clusters with $I_C$ values smaller than 0.2 as *upstream* clusters and the clusters with $I_C$ values between those two thresholds as *core* clusters.

**Fig. 4:**
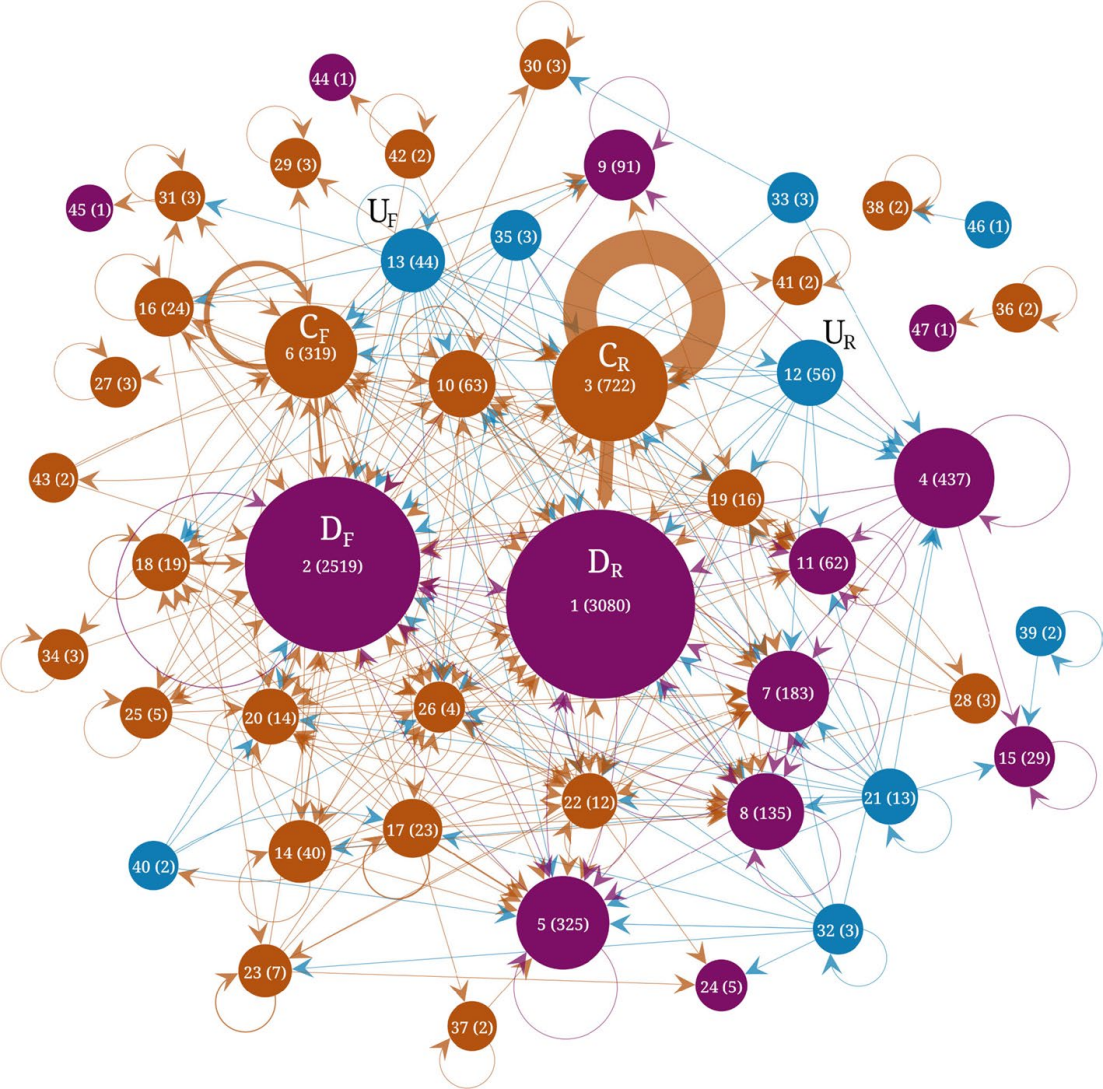
Flow stability clustering of the telegram channels network $G_1$ (Sep. 2015 to June 2019). Nodes represent the communities and edges represent the number of links between channels in each community. Upstream communities are shown in blue, core communities in brown and downstream communities in purple. The labels indicate the rank of each community in terms of size and the size is indicated in parenthesis

**Fig. 5:**
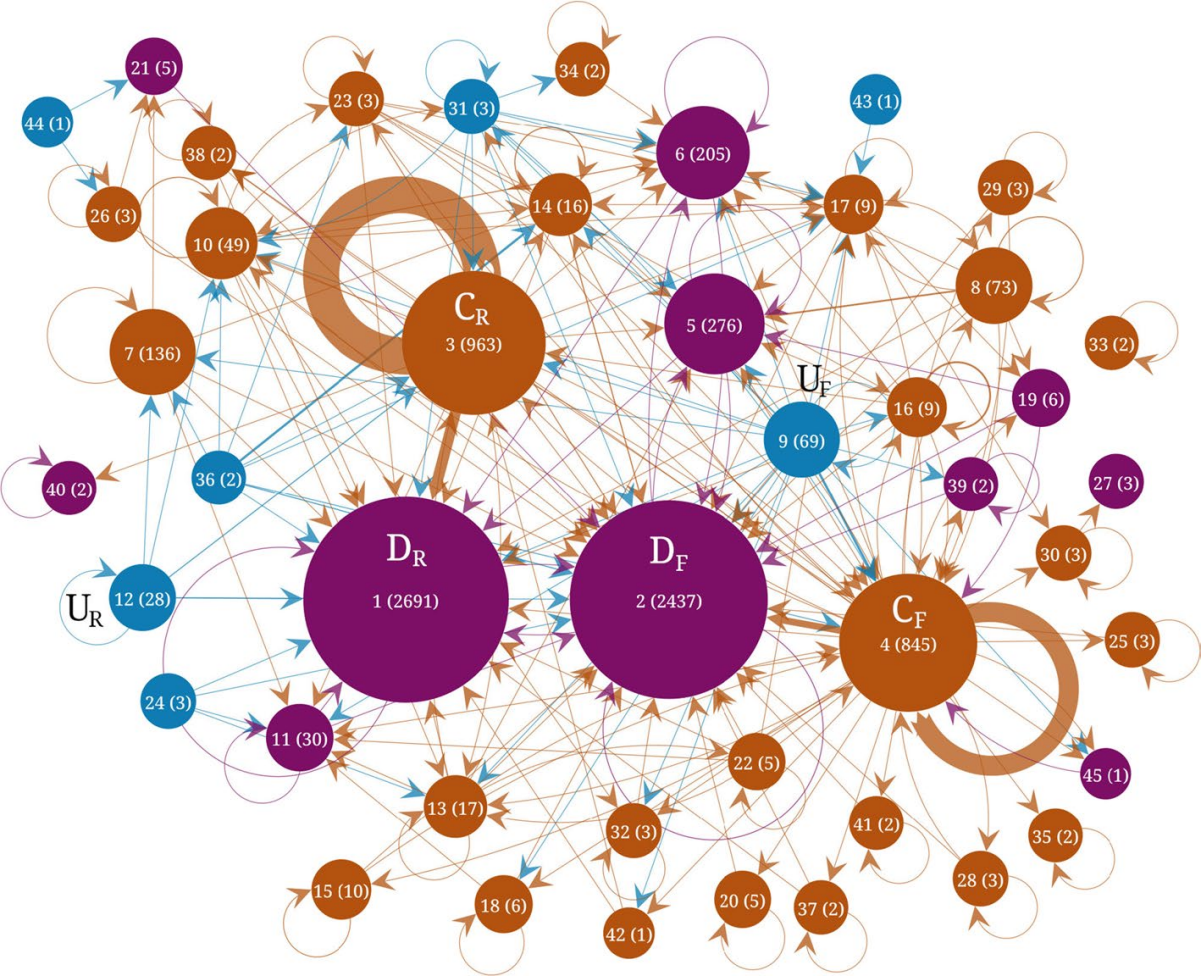
Flow stability clustering of the telegram channels network $G_2$ (July 2019 to Feb. 2020). Nodes represent the communities and edges represents the number of links between channels in each community. Upstream communities are shown in blue, core communities in brown and downstream communities in purple. The labels indicate the rank of each community in terms of size and the size is indicated in parenthesis

**Fig. 6:**
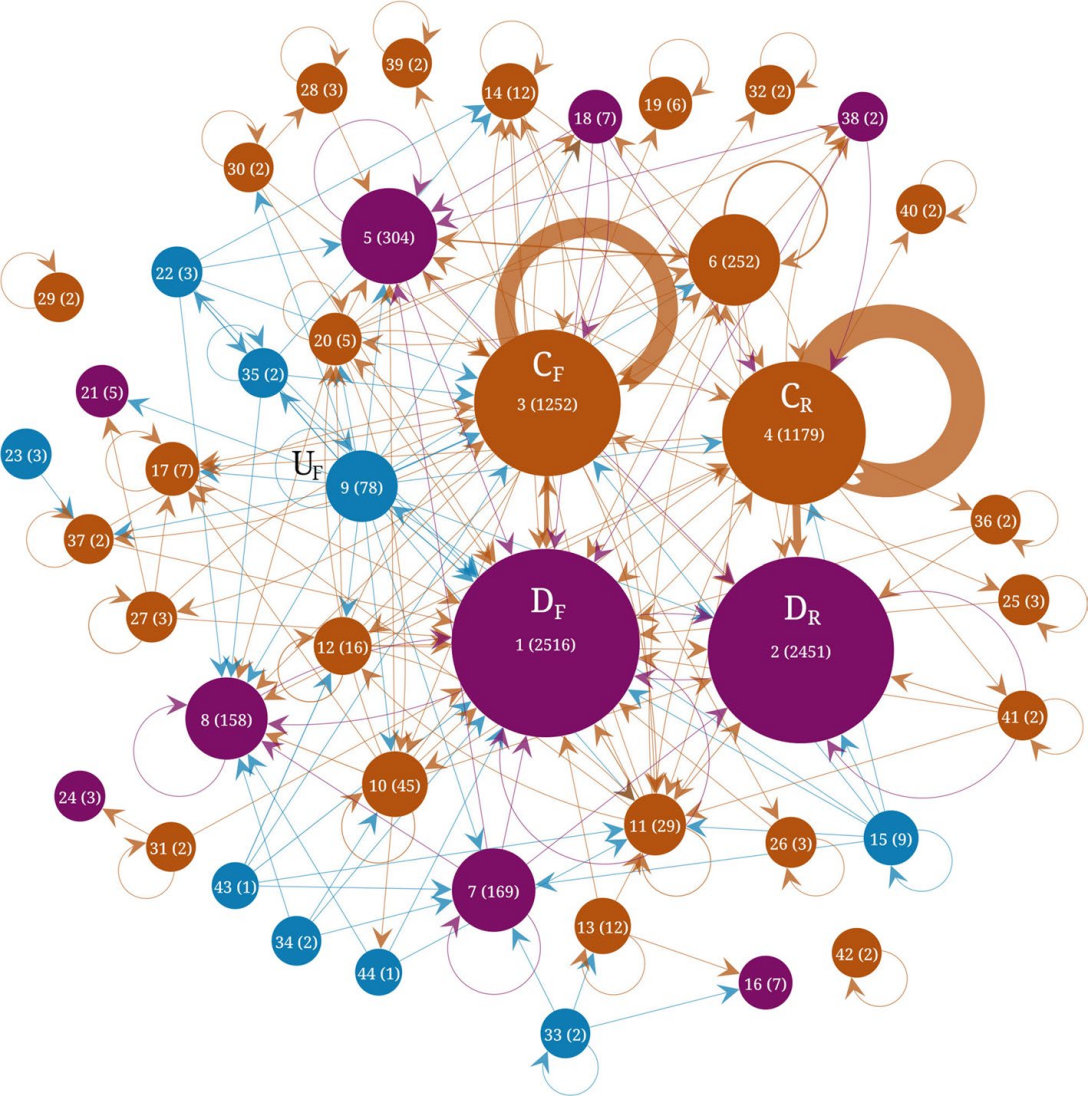
Flow stability clustering of the telegram channels network $G_3$ (March 2020 to Dec. 2020). Nodes represent the communities and edges represents the number of links between channels in each community. Upstream communities are shown in blue, core communities in brown and downstream communities in purple. The labels indicate the rank of each community in terms of size and the size is indicated in parenthesis

Figures 4, 5 and 6 show the results of the flow stability clustering applied to the three Telegram networks. Each community is displayed as a node with a size proportional to the size of the community. Edges represent the number of links between the communities, with self-loops representing the number of internal links. Core communities tend to have the largest self-loops. The structure of the three networks is characterized by the presence of two main core communities and two main downstream communities labeled $C_F, C_R, D_F, \& \, D_R$, respectively. The rank, in terms of size, is shown in parentheses on each community together with its size. The two main core and two main downstream communities are arranged in two pairs. Each of these pairs can be understood as formed by one core community where channels mention each other frequently and a downstream community with channels that are frequently mentioned in the core community but that do not reciprocate these interactions. The downstream communities are rather the object of the discussions. The upstream communities are comparatively small which may be due in part to the collection method that necessarily follows downstream links between channels.

### Channels

We identify the most important channels by ranking them using their Katz centrality (Katz 1953). The Katz centrality of node *i* is the weighted sum of all walks emanating from *i*, with the count for walks of length $\ell$ weighted by a factor $\alpha ^\ell$ where 0 $<\alpha<$ 1. A walk in a network is an alternating sequence of nodes and edges in which every node is incident to both the edges that come before and after it in the sequence (Bovet and Makse 2021). Here, walks represent the potential trajectories of users in the Telegram network. The Katz centrality captures long-range structures in the network, but the importance of longer walks is diminished compared to shorter walks. Here we choose $\alpha =0.99 \cdot 1/\lambda _1$ where $\lambda _1$ is the largest eigenvalue of the adjacency matrix.

We rank channels in the core and downstream communities using their in-Katz, centrality, i.e. channels that are more highly linked by other channels are more highly ranked. In the upstream communities, we are interested to find channels that serve as entry points to the network and we, therefore, use their out-Katz centrality, i.e. highly ranked channels are channels that are the starting point of many walks in the network. We display the names of channels belonging to organizations, news commentators and public figures. Channels with names potentially corresponding to private individuals are replaced by ### to respect privacy (we count only two such channels in the top ranks).

Table 2 shows the top channels for the first network covering Sep. 2015 to June 2019. The top channels in the largest downstream community ($D_R$) are mainly Russian-language broadcast channels about Russian political and business news. Only two channels are affiliated with public entities. TJournal is the official channel of the news aggregator tjournal.ru and varlamov is the official channel of the blogger Ilya Varlamov (varlamov.ru). The other top channels do not directly state the identities of their owners. Top channels in the second largest downstream community ($D_F$) are linked to the U.S. president Donald Trump (realdonaldtrump, WhiteHouse, POTUS), however, these channels are not officially recognized and have almost no activity. They are highly linked from other channels probably because Donald Trump was at the center of many discussions, but not for their content. Other top channels in this community include news-related channels: breaking911 (breaking911.com) is a U.S. news website flagged for publishing very low factual content by the website Media Bias Fact Check (MBFC),^5^ jahan_24 is a channel publishing news from the Islamic Republic of Iran News Network (iribnews.ir) in Persian, ReutersWorldChannel is a channel broadcasting from reuters.com but is not officially affiliated with Reuters. The channel foxnews is also not the official channel of the cable news TV channel FoxNews and is not active. The rest of the top channels are mainly about memes and broadcasting far-right ideologies. The top channels in the third largest downstream community include news aggregator channels in Spanish (Redes7, Noticias2018), Persian (vatankhahan), Brazilian (criticanacional linked to criticanacional.com.br, BrasilSurreal) and Arabic (almubeen102). Other channels include HA_alshami02 linked to a military faction affiliated with the Turkish-backed Free Syrian Army (in Arabic), PatriotasConservadores promoting Brazilian right-wing ideologies, OlavoTemRazaoCanal is linked to a Brazilian far-right conspiracy theorist^6^ and red_color that broadcasts alerts of imminent attacks by rockets in Israeli settlements around Gaza.^7^

**Table 2:** Most important channels in the main downstream, core and upstream clusters for the network $G_1$ in Fig. 4 corresponding to the period Sep. 2015 to June 2019

Size rank	Top channels in downstream communities
1 ($D_R$)	kremlin_mother_expert, sexy_moscow, solarstorm, TJournal, gorod095, nebrexnya, justthejudge, nourlnews, varlamov, pro_IT_2018
2 ($D_F$)	realdonaldtrump, WhiteHouse, breaking911, jahan_24, POTUS, ancapistantm, ReutersWorldChannel, YLYL4chan, dailyredpill, XDProductions, DieForIsraelGoy, AnarchoMemes, AltMemes, khamenei_ir, anarchiaautonomia, animach, PoliticalMemes, DanScavino, foxnews, roopab
4	Redes7, OlavoTemRazaoCanal, Noticias2018, ###, HA_alshami02, criticanacional, PatriotasConservadores, almubeen102, red_color, BrasilSurreal

Size rank	Top channels in core communities
3 ($C_R$)	go338, karaulny, kbrvdvkr, rt_russian, stormdaily, bbbreaking, kononenkome, operdrain, tv360ru, mediatech
6 ($C_F$)	randomanonch, Thecelticempire, WhiteIsRight, sgmeme, BloodAndHonour, NazBol, CIGtelegram, beadymanor, MiloOfficial, shitpost, toalibertarian, TommyRobinsonNews, pjwnews, pol_4chan, HansTerrorwave, officialmilo, loomeredofficial, AntifaPublicWatch, V_of_Europe*, JackDawkins
10	just_hmmm, r_unexpected, programmer_humor, get_happiness, awwnime, r_behindthegifs, PoliticalHumor, r_Showerthoughts, r_dankmemes, r_HighQualityGifs

Size rank	Top channels in upstream communities
12 ($U_R$)	severnygorod, live_kuban, vampov, ErnestMakarenko, gorodbratsk, ci_newsblock, na_rajone, ordynets, sevenandmedia, lubiczonline
13 ($U_F$)	LeHumbleKekVerse*, contraototalitarismodaonuenom, brexiteerschatlounge*, judenpresse_archive, q_anons*, CrypticCoinVIP*, fitinorfuckoff*, trrchat*, Onehundredfags*, KingdomZombe*, News_cabinet_news, bannedforlife, IB_MeckPomm, tehmoonwalkers*, thecarnalconservative, SundayLongLive*, childrenmatter*, letzcrowd, DieWahrheitnurdieWahrheit, ###

We give the top channels ranked by their in-Katz centrality for channels in the downstream and core clusters and ranked by their out-Katz centrality in the upstream clusters. We show the top 20 channels, sorted according to their Katz score, in communities linked to the UK and US far-right and the top 10 channels in other communities. Channels marked with an asterisk correspond to group chats while the other ones correspond to broadcast channels

Top channels in the largest core community ($C_R$, Table 2) are all in the Russian language. They include channels commenting on Russian news and politics (go338, karaulny, kbrvdvkr, bbbreaking, operdrain and mediatech) that do not explicitly reveal the identities of their owners. Channels related to Russian media outlets are also present: rt_russian (a state-controlled international television network funded by the Russian government:^8^ russian.rt.com), stormdaily (dailystorm.ru) and tv360ru (360tv.ru). The channel kononenkome is related to the political activist Maksim Kononenko.^9^ Top channels in the second largest core community ($C_F$) include channels that link to content from the anonymous “Politically Incorrect” discussion imageboard 4chan.org/pol/ (randomanonch and pol_4chan) that frequently contains racist, white supremacist, antisemitic, islamophobic, misogynistic, and anti-LGBT themes (Hine et al. 2017; Merrin 2019; Baele et al. 2021). Several channels have names related to white supremacist themes (e.g. Thecelticempire or WhiteIsRight) or channel information that explicitly relates to far-right themes. CIGtelegram “presents viewers a controversial blend of ultraright genopolitics” according to its channel information. Nazbol is about National Bolshevism, a radical political movement that combines ultranationalism and communism.^10^ AntifaPublicWatch is an anti-Antifa channel. HansTerrorwave has been reported to have been used by white supremacists to organize violence.^11^ Several channels are associated with British far-right personalities: Milo Yiannopoulos^12^ (MiloOfficial), Tommy Robinson^13^ (TommyRobinsonNews), Paul Joseph Watson^14^ (pjwnews). The channel loomeredofficial is associated with the American far-right personality Laura Loomer.^15^ The channels toalibertarian and shitpost are channels broadcasting memes. The only group chat in the top 20 channels is V_of_Europe which is related to the news website Voice of Europe that ceased to operate and was flagged as disseminating extreme right propaganda and conspiracy theories by MBFC.^16^ The top channels in the third largest core community are channels that broadcast posts from various “subbreddits”, i.e. communities on the website reddit.com.

In the largest upstream community ($U_R$, Table 2), all top channels are in Russian language. The top channels provide independent commenting about Russian local news (severnygorod, live_kuban, gorodbratsk, na_rajone, sevenandmedia, lubiczonline) or broadcast memes (vampov). The channel ci_newsblock claims to post about competitive intelligence and data leaks and the channel ErnestMakarenko belongs to a Moscow councilor.^17^ The second largest upstream community ($U_F$) contains the most group chats in the top channels. Most of the group chats are for discussions about themes linked to right-wing politics (LeHumbleKekVerse, brexiteerschatlounge), conspiracy theories (q_anons) and extreme right hateful ideologies (fitinorfuckoff, trrchat, Onehundredfags, KingdomZombe). All the top channels are in English, except for contraototalitarismodaonuenom which is in Brazilian and related to the Brazilian President Jair Bolsonaro, News_cabinet_news which is in Hebrew and provides real-time field reports and security events from Gaza Strip, Judea, Lebanon, Syria, Iraq and Iran, and DieWahrheitnurdieWahrheit which is in German (The truth and only the truth).

**Table 3:** Most important channels in the main downstream, core and upstream clusters for the network $G_2$ in Fig. 5 corresponding to the period July 2019 to Feb. 2020

Size rank	Top channels in downstream communities
1 ($D_R$)	Uzynqulaq, dinamika_ria, MakarenkoLive, eshnic, antizlobinlive, sexy_moscow, sevkav, TJournal, Buddykiton, nourlnews
2 ($D_F$)	realdonaldtrump, breaking911, WhiteHouse, InfowarsNews, durov, Notactuallyfunny, TrumpWarRoom, PS752, ReutersWorldChannel, Breitbart, realtimenewsbroadcasts, JoeBiden, hongkongfp, DonaldJTrumpJr, SvenLiebichChat*, telegram, southfronteng, foxnews, AnarchoMemes, anarchiaautonomia
5	r_privacy, reddit_android, programmer_humor, manutd, just_hmmm, r_gifs, soccer_reddit, r_libertarian, r_WikiLeaks, rfurryirl

Size rank	Top channels in core communities
3 ($C_R$)	rt_russian, bbbreaking, SolovievLive, margaritasimonyan, SIL0VIKI, karaulny, rlz_the_kraken, sashakots, youlistenedmayak, kremlinprachka
4 ($C_F$)	AntifaPublicWatch, JackDawkins, BritainFirst, TommyRobinsonNews, vinniesullivan, police_frequency, MiloOfficial, Brexiteers, therealityreport, luketrrcage, PatriotNewz, leegarrettupdates, dannytommo, trump, JaydaFransen, loomeredofficial, newspanopticon, MARIOBORG20, Jaydafransensupporters, RedDog71Media
7	italia24hnews, WTF_Radio, coindesk_news, Partisan2015, HowToFind, Arteshban, LiverpoolFCNews, IrVatan, LoyalistsHQ, HA_alshami02

Size rank	Top channels in upstream communities
9 ($U_F$)	KingdomZombe*, TheVaultVids*, nomoremosqueschat*, PatrioticGroupChat*, AfrikanerBoer, nomorelockdown*, BritPatriots*, ChronoClockCorp*, realDonaldjTrump, loomeredchat*, BritPol1*, infowarsofficalchat*, SundayLongLive*, UKIPSUPPORTERS*, Excarkun, GalliaArchive, TheVaultTommyRobinsonWeAreTommy*, students4trump*, ultracatolico*, GroomingGangsPredatorAwareness*
12 ($U_R$)	agrgtr, armnewz, SkuratoffOne, skrepka2020, ci_newsblock, vampov, snimulapshu, bosphorus1861, bgmos, W_IN_FIRE

We give the top channels ranked by their in-Katz centrality for channels in the downstream and core clusters and ranked by their out-Katz centrality in the upstream clusters. We show the top 20 channels, sorted according to their Katz score, in communities linked to the UK and US far-right and the top 10 channels in other communities. Channels marked with an asterisk correspond to group chats while the other ones correspond to broadcast channels

Table 3 shows the top channels in the main downstream, core and upstream communities for the second network covering the period from July 2019 to February 2020. The two largest downstream communities have several similar top channels than in the first network ($D_R$ and $D_F$, Table 2), with Russian-language channels in the largest and English language channels in the second largest. Several new channels appeared in the top ranks such as InfowarsNews and Breitbart, linked to the American far-right news websites infowars.com, owned by Alex Jones, and breitbart.com, whose former executive chairman was Steve Bannon. Info Wars News has been flagged for disseminating conspiracy theories and fake news (Kaiser et al. 2020; Chong 2019) and Breitbart News has been rated as a questionable source with an extreme right bias by MBFC.^18^ A group chat in German, SvenLiebichChat, about a right-wing extremist^19^ also appeared in the top channels of this community. We also see a number of new channels that, although other channels highly link to them, have almost no activity and are not official accounts (e.g. TrumpWarRoom, JoeBiden, DonaldJTrumpJr, foxnews) indicating that they are used mostly because they are at the center of discussions in the core communities. They use the same name than their official counterpart in Twitter. The list of top channels in the third largest downstream community contains channels linking to the website reddit.com with many channels in the first network's third largest core community.

The two largest core communities ($C_R$ and $C_F$, Table 3) also share several top channels with the top channels of the two largest core communities of the previous network (Table 2). The channel of Margarita Simonyan (margaritasimonyan), editor-in-chief of RT,^20^ appeared in the top channels of the $C_R$ community. New channels in the second largest core community include the BritianFirst broadcast channel and channels of various personalities of the UK far-right. The third largest core community is a relatively small community (137 channels, see Fig. 5) with an assortment of diverse broadcast channels with unofficial commentary on local news, cryptocurrencies or football. Similarly to the core communities, the two largest upstream communities share several top channels with the two largest upstream communities of the first network (Table 2). The community with many group chats related to the far-right is now the largest and we see several new group chats. One of the group chats is named nomorelockdown referring to the lockdowns imposed during the COVID19 pandemic that started after the time range used to construct this network. This could be explained by the fact that this channel changed its name when the pandemic started.

**Table 4:** Most important channels in the main downstream, core and upstream clusters for the network $G_3$ in Fig. 6 corresponding to the period March 2020 to Dec. 2020

Size rank	Top channels in downstream communities
1 ($D_F$)	newspanopticon, SidneyPowell1, The_Duran, eQsynews, realdonaldtrump, leegarrettupdates, Breitbart, The_Library_II, ProudBoysOfficial, felixrex, yellowvest, patriotictalk*, TrTalkChris, JoleneBuntingUK, nationisviribus, forbiddenbookclub, Altnewsnetwork, project_veritas, zerohedge, WhiteHouse
2 ($D_R$)	marochkolive, kremlin_mother_expert, Uzynqulaq, tass_agency, uranews, rucriminalinfo, crimeainform, ykt2100, emphasises, borusio
5	r_interestingasfuck, just_hmmm, rfurryirl, r_imaginary_network, r_movies, MemeArea, r_rupaulsdragrace, r_evilbuildings, r_porn, R_Blursedimages

Size rank	Top channels in core communities
3 ($C_F$)	therealityreport, vinniesullivan, exposingculturalmarxism, usvoterfraud, TRRMEDIA, RealVincentJames, UKSTREETCRIME, Liberal_Maniacal_Psychosis_Arc, luketrrcage, politicsdebate*, AlSangmoore, westcoastintel, WesternHeritage, BellumActaNews, therightwinggroup*, trrchat*, OKGroomerMunitionsFactory, AntifaPublicWatch, vinniesullivanchat*, jamesgoddard89
4 ($C_R$)	bbbreaking, rt_russian, rian_ru, russica2, kremlebezBashennik, dimsmirnov175, kaktovottak, sputniklive, sashakots, master_pera
6	r_WikiLeaks, COMPLETE_ANARCHY, r_mapporn, r_channels*, reddit2telegram, admeme, r_Damnthatsinteresting, r_privacy, programmer_humor, tyingherhairup

Size rank	Top channels in upstream communities
9 ($U_F$)	steetcrimechat*, donaldtrumpchat*, aviyeminichats*, FuckYourFeels*, fitinorfuckoff*, magafirstnews*, ResistMarxismPublic*, RDBAthiests*, TrumpTime, socialismbar*, x22chat*, ###, TheVaultVids*, GFR_Henderson, LeHumbleKekVerse*, PatrioticGroupChat*, catholicwignat*, AnarchoCapitalism*, NEWSWORTHY1*, students4trump*
15	YouHaveToKnow, Right_Europe*, firstnose, HaghNewss, Tellmama, NavalAcademy*, forgeeks, alsyasehnews, Aramcaramba

We give the top channels ranked by their in-Katz centrality for channels in the downstream and core clusters and ranked by their out-Katz centrality in the upstream clusters. We show the top 20 channels, sorted according to their Katz score, in communities linked to the UK and US far-right and the top 10 channels in other communities. Channels marked with an asterisk correspond to group chats while the other ones correspond to broadcast channels

Table 4 shows the top channels in the main communities for the period of March 2020 to December 2020. The lists of top channels in the three largest downstream communities have a large overlap with the same communities of the previous network. In the $D_F$ community, we see the addition of new alternative news channels (e.g. newspanopticon, eQsynews) and channels related to right-wing personalities such as SidneyPowell1, associated with the American attorney, former federal prosecutor, and conspiracy theorist Sidney Katherine Powell^21^ or JoleneBuntingUK, linked to Jolene Bunting, a former Belfast councilor associated with Britain First.^22^ We also see the channel ProudBoysOfficial of the homonymous American far-right, neo-fascist, organization that became popular during the first U.S. presidential debate in 2020.^23^

The top channels in the main core communities (Table 4) also overlap with the top channels in the same communities of the previous network (Table 3). The community containing U.K. and U.S. far-right channels ($C_F$) is now larger than the one containing Russian channels. New addition includes a channel linked to the 2020 U.S. presidential elections (usvoterfraud) and a group chat that was previously in the upstream community (trrchat linked to “The Reality Report”). Three group chats are now present in the top 20 channels of the largest core community indicating that they not only link to other channels, i.e. comment on the content of other channels, but are also linked to by other channels of the core community. This reveals their more central role compared to group chats in the downstream communities. The channel sputniklive appears in the top channels of the second largest core community ($C_R$). The Russian state-owned news agency Sputnik News, together with RT (channel rt_russian) have been described as part of the propaganda apparatus of the Russian government and frequent sources of pro-Kremlin disinformation (Karlsen 2016; Benkler et al. 2018; Ižak 2019). The top channels in the two largest upstream communities show a large turnover. In the largest community, 15 channels appear in the top 20 for the first time. Several new group chats are linked to Donald Trump (donaldtrumpchat, FuckYourFeels, magafirstnews).

### Network evolution

**Fig. 7:**
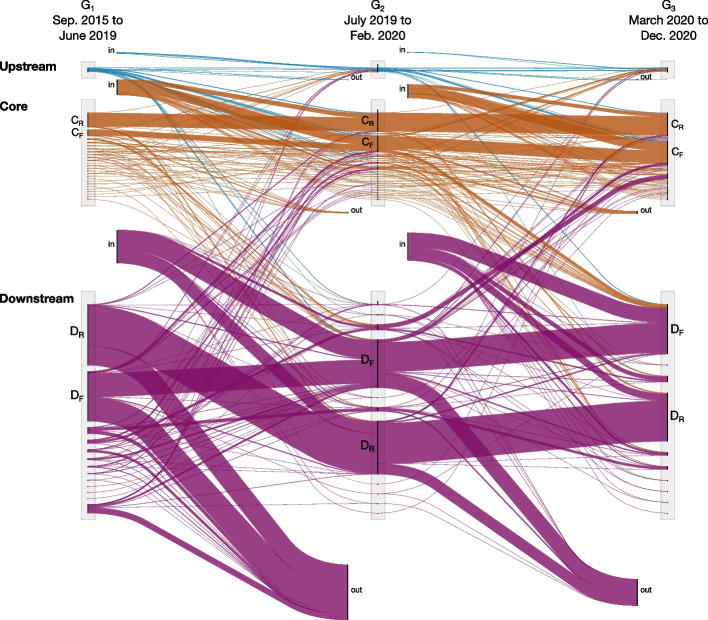
Alluvial diagram of the evolution of the communities across the three periods. Flows represent the movement of channels from community to community across time. New channels entering and existing in the system are represented as arriving from “in” and “out” groups, respectively. The upstream, core and downstream communities are grouped in three different bundles and shown in different colors

Figure 7 shows the evolution of the communities from network to network as an alluvial diagram. The flows in the figure represent the movement of channels from the communities of one network to the following. The incoming and outgoing flows represent the number of new channels entering the system and channels exiting the system between the different time windows. We label with a $C_R$ the flow corresponding to the core community containing the most top channels linked to the far-right movements English-speaking countries (see also Figs. 4, 5, 6). We see that, across time, this community is persistent and continuously growing, going from 6th to 3rd in terms of size, with 319 channels in $G_1$, 845 in $G_2$ and 1252 in $G_3$. We also notice that the channels joining this community are majoritarily coming from the exterior, meaning that they are either new or coming from outside the system. We also see very few channels going out of the system, which happens when a channel closes, stops showing activity or becomes private. Between $G_1$ and $G_2$, 13 channels from this community exit the system and 438 join the community from outside the system. Between $G_2$ and $G_3$, the same analysis gives 54 channels exiting and 465 entering. The core community with top channels including Russian news media and commentary is labeled as $C_R$. As for the far-right community ($C_F$), this community is growing with mostly new channels joining it and only a small fraction leaving it during each period.

The downstream community having top channels in Russian language is labeled with a $D_R$ in Fig. 7. The downstream community containing right-wing news channels and far-right related channels is labeled with a $D_F$. We see that, contrary to the situation with the core communities, a large fraction of the channels in these communities change between each period. Between $G_1$ and $G_2$, 988 channels from $D_R$ and 1187 channels from $D_F$ exit the system while 593 new channels join $D_R$ and 928 new channels join $D_F$. Meanwhile, $D_R$ shrank from 3080 to 2691 channels and $D_F$ grew from 2519 to 2691 channels. Between $G_2$ and $G_3$, 539 channels from $D_R$ and 722 channels from $D_F$ exit the system while 336 new channels join $D_R$ and 737 new channels join $D_F$. In $G_3$, $D_R$ has 2452 channels and $D_F$ has 2516 channels.

**Table 5:** Movement of channels across upstream, core and downstream communities between the networks corresponding to different time periods

*G*_1_ to *G*_2_	Upstream (%)	Core (%)	Downstream (%)	Exterior (%)
Upstream	13	66	13	8
Core	2	83	11	4
Downstream	0.1	3	56	41

*G*_2_ to *G*_3_	Upstream (%)	Core (%)	Downstream (%)	Exterior (%)
Upstream	17	68	6	9
Core	3	78	13	6
Downstream	0.2	7	69	24

The fraction moving to the exterior corresponds to the fraction of channels exiting the system

Figure 7 also reveals that only a limited number of channels move between the upstream, core and downstream groups. We report the fraction of channels moving across community types and exiting the system in Table 5. We see that, apart for upstream channels that, for the most part, move to the core, channels in the core and in the downstream communities remain, for the most part, in the same category of community. The exchanges between core and downstream are limited (maximum of 13% from core to downstream and 7% from downstream to core).

These differences in dynamics between upstream, core and downstream communities indicate that channels in different groups play different roles in this system. The large renewal of channels in the downstream communities suggests that these channels change according to the change of focus of the discussions happening in the core and upstream communities. The core communities represent the center of the network which slowly grows with time. They contain channels that refer to each other and provide commentary about channels in the downstream networks. The upstream communities seem to be entry points in the network where most discussions between users happen. A large fraction of these channels eventually moves to the core as they become more popular and are also referred to by other channels in the core.

### Relations to external websites

In order to better understand how channels in different communities relate to external websites, we extract all the URLs (Uniform Resource Locator) that are shared in the main communities of each network. We resolve URLs that use a URL shortening service (i.e. bitly.com) (Yin 2018) and match popular custom-shortened URLs to their full domain name (i.e. nyti.ms to nytimes.com) to extract the domain name of each link. Tables 6, 7 and 8 list the top 20 domain names shared in the main communities of the three networks.

To evaluate the change in the ranked domain lists across networks, we compute the Rank-Biased Overlap similarity (RBO) (Webber et al. 2010) between the lists obtained in the first, second and third networks (see Table 9). The RBO measures of the similarity between incomplete rankings, handles non-conjointness and weights high ranks more heavily than low ones. Such properties are desirable here because the lists of domains are extensive but we are mostly interested in the variation in of most popular domains and because domain names can appear or disappear from one time frame to the next. We chose the weighting parameter $p=0.91$, giving 95% of the weight to the top 20 elements of the lists.

**Table 6:** Top URL domains shared in the main communities of the network $G_1$ (Sep. 2015 to June 2019, Fig. 4)

	Downstream $D_R$		Downstream $D_F$		Core $C_R$	
	Domain	Count	Domain	Count	Domain	Count
1	rossaprimavera.ru	136,822	nytimes.com	85,992	lenta.ru	171,246
2	readhacker.news	30,768	twitter.com	81,016	telegraph.co.uk	119,665
3	news.ycombinator.com	14,899	zerohedge.com	63,991	youtube.com	75,275
4	hromadske.ua	9059	dailycaller.com	54,614	twitter.com	63,790
5	tass.ru	8298	news.ycombinator.com	54,519	meduza.io	52,747
6	vognebroda.net	7619	breitbart.com	47,563	ntv.ru	44,414
7	spzh.news	6835	bbc.co.uk	39,227	riafan.ru	43,224
8	nashaniva.com	5573	reuters.com	22,969	reddit.com	26,284
9	telegraph.co.uk	5415	iribnews.ir	19,305	ria.ru	24,157
10	rbc.ru	4738	youtube.com	11,299	vk.com	23,987
11	uoj.org.ua	4364	southfront.org	8651	facebook.com	20,578
12	inosmi.ru	3907	smokeroom.com	6011	tvrain.ru	20,539
13	warspot.ru	2798	infowars.com	5730	rt.com	18,322
14	mobile-review.com	2470	en.wikipedia.org	4730	znak.com	17,452
15	kp.by	1772	whatsapp.net	4249	kp.ru	17,153
16	github.com	1685	washingtonpost.com	3719	kommersant.ru	13,895
17	youtube.com	1156	reddit.com	3607	vz.ru	13,330
18	vk.cc	1011	orthochristian.com	3087	rbc.ru	12,160
19	nytimes.com	1003	github.com	2705	iz.ru	12,137
20	twitter.com	848	russia-insider.com	2699	govoritmoskva.ru	12,018

	Core $C_F$		Upstream $U_R$		Upstream $U_F$	
	Domain	Count	Domain	Count	Domain	Count
1	twitter.com	343,326	ru.sputnik.kg	37,962	youtube.com	13,909
2	4chan.org	94,314	bst.bratsk.ru	3905	twitter.com	12,629
3	youtube.com	51,131	twitter.com	3640	infowars.com	2951
4	voiceofeurope.com	41,546	bratsk-city.ru	3176	facebook.com	1948
5	reddit.com	40,754	topspb.tv	2795	usatoday.com	936
6	sputniknews.com	21,037	medium.com	2669	medium.com	923
7	zerohedge.com	19,136	livekuban.ru	2337	renovamidia.com.br	845
8	rt.com	16,393	youtube.com	2313	reddit.com	843
9	almasdarnews.com	13,953	blog.ins.world	1943	breitbart.com	734
10	dailystormer.name	10,192	uiamp.org.ua	1448	telegraph.co.uk	688
11	bloomberg.com	6298	zn38.ru	1386	cointelegraph.com	666
12	euronews.com	5942	telegraph.co.uk	1130	crypticcoin.io	662
13	marketwatch.com	4729	tokensale.ins.world	1121	brighteon.com	594
14	southfront.org	4322	ins.world	1105	instagram.com	427
15	casoaislado.com	4287	boon.vc	1075	images.cointelegraph.com	381
16	telegraph.co.uk	3041	tkgorod.ru	1042	criticanacional.com.br	349
17	archive.is	2815	reddit.com	808	ccn.com	333
18	dailymail.co.uk	2695	belaruspartisan.by	806	ctlgr.com	330
19	hindustantimes.com	2619	github.com	648	zerohedge.com	310
20	archive.fo	2585	gate.io	599	cumta.morhaviv.com	288

The top domain names in the Russian downstream community ($D_R$) for the period of Sep. 2015 to June 2019 include several news websites (Table 6). The top one, with approximately 4.5 times more counts than the second one, is the Russian news website rossaprimavera.ru. Its description on its Twitter page,^24^ reads “Our movement fights for #Humanity #Communism”. This website has been flagged as spreading pro-Russian disinformation by the flagship project of the European External Action Service’s East StratCom Task Force EuvsDisinfo.^25^ Its position at the top is explained by the presence of the channel rossaprimavera in this community which regularly links to articles of its website. The second and third domain names are both related to the website Hacker News which is a social news website, in English, focusing on computer science and entrepreneurship. In the remaining top 20 domain names, we see mainly Russian and Ukrainian news websites. The only social media website, Twitter, is in the last position. The domain name vk.cc is related to the Russian social media VKontakte, but is linked to their URL shortening service and appears here due to a number of shortened URLs that we were not able to resolve.

The top domains in the far-right related downstream cluster ($D_F$) also contain a large proportion of news websites, but mainly from the US and UK. The exception is the Iranian news website iribnews.ir. We see a mix of well-known center/left-leaning news outlets (e.g. nytimes.com, bbc.co.uk, whasingtonpost.com) and newer right-wing/far-right news outlets (e.g. zerohedge.com, dailycaller.com, breitbart.com, infowars.com). We also see two websites that have been classified as sources with low factual reporting, diffusing conspiracy theories and Russian propaganda by MBFC: southfront.org^26^ and russia-insider.com.^27^ Social media websites are also present in the top domain names: Twitter, Youtube, Whatsapp, Reddit.

In the Russian core community ($C_R$), we see different websites than in the Russian downstream community. The two most popular websites are the Russian online newspaper lenta.ru and the British newspaper telegraph.co.uk. Other websites in the top 20 include meduza.io, a news website founded by former employees of lenta.ru which is based in Lativa in order to escape censorship from the Russian government,^28^ntv.ru a television channel controlled by GazProm Media since 2001,^29^ and several news outlets that have been flagged as spreading pro-Russian disinformation by EuvsDisinfo:^30^riafan.ru, ria.ru, rt.com, znak.com, kp.ru, kommersant.ru, vz.ru, rbc.ru, iz.ru, govoritmoskva.ru. Contrary to the Russian downstream community, several social media websites are also present in the top domain names of the Russian core community: YouTube, Twitter, Reddit, VKontakte and Facebook.

The far-right core community ($C_F$) also contains several social media websites in the top 20 domain names with Twitter, 4chan and Youtube being the top three shared domain names. We also see far-right news outlets such as Voice of Europe, Zero Hedge^31^ and The Daily Stormer.^32^ The international Kremlin-controlled news outlets sputniknews.com and rt.com are also present.

The branch antenna of the Sputnik News online website, in Russian, is the most shared domain name in the Russian upstream community. Other top domain names in this community include local television channels and news outlets, blogs, a Ukrainian political think tank (uiamp.org.ua) and social media websites (Twitter, YouTube and Reddit).

In the far-right upstream community ($U_F$) social media YouTube and Twitter are the most shared websites by a large margin. Other top websites include American far-right news websites and more traditional American center news outlets (usatoday.com and cnn.com). The blog hosting website medium.com is also present.

**Table 7:** Top URL domains shared in the main communities of the network $G_2$ (July. 2019 to Feb. 2020, Fig. 5)

	Downstream $D_R$		Downstream $D_F$		Core $C_R$	
	Domain	Count	Domain	Count	Domain	Count
1	rossaprimavera.ru	74,544	twitter.com	71,855	telegraph.co.uk	57,569
2	lenta.ru	34,247	nytimes.com	22,168	youtube.com	44,505
3	tasnimnews.com	18,008	breitbart.com	21,788	twitter.com	12,893
4	meduza.io	5564	farsnews.ir	20,661	facebook.com	11,972
5	rt.com	3825	cnn.com	20,338	rbc.ru	10,889
6	news.ru	2991	readhacker.news	19,639	govoritmoskva.ru	10,657
7	spzh.news	2942	dailycaller.com	17,944	znak.com	10,551
8	mbk-news.appspot.com	2904	news.ycombinator.com	15,670	instagram.com	10,475
9	republic.ru	1547	bbc.co.uk	14,525	kommersant.ru	8579
10	news.tut.by	1513	zerohedge.com	13,326	meduza.io	8076
11	warspot.ru	1497	reddit.com	10,571	ria.ru	7858
12	nashaniva.com	1294	nitter.pro	10,340	vk.com	7789
13	svpressa.ru	1091	ift.tt	8534	tvrain.ru	7600
14	telegraph.co.uk	1061	youtube.com	8399	iarex.ru	6753
15	youtube.com	604	infowars.com	7819	tass.ru	6393
16	targetingsstorage.blob.core.windows.net	584	epochtimes.de	6096	forbes.ru	5941
17	seance.ru	518	southfront.org	4613	kp.ru	5754
18	mobile-review.com	517	palinfo.com	2760	mskagency.ru	5526
19	gorky.media	512	timesofindia.indiatimes.com	2463	hromadske.ua	5489
20	vk.com	300	fort-russ.com	2217	riafan.ru	5379

	Core $C_F$		Upstream $U_F$		Upstream $U_R$	
	Domain	Count	Domain	Count	Domain	Count
1	twitter.com	771,700	youtube.com	11,572	telegraph.co.uk	1362
2	youtube.com	156,516	twitter.com	4436	kuban.kp.ru	906
3	reddit.com	52,229	jaydafransen.online	746	belaruspartisan.by	528
4	4chan.org	51,888	facebook.com	707	armenia-news24.ru	485
5	sputniknews.com	16,123	bitchute.com	647	youtube.com	398
6	infowars.com	15,387	worldunity.me	636	nalog.ru	249
7	zerohedge.com	14,981	telegraph.co.uk	581	twitter.com	235
8	voiceofeurope.com	14,704	breitbart.com	378	consultant.ru	205
9	rt.com	12,546	dailymail.co.uk	334	medium.com	202
10	yna.kr	10,458	infowars.com	301	klerk.ru	201
11	telegraph.co.uk	9301	ct.com	254	swipe.io	136
12	almasdarnews.com	9127	businesstech.co.za	246	kommersant.ru	128
13	archive.is	7951	images.cointelegraph.com	236	rbc.ru	127
14	hindustantimes.com	7800	instagram.com	204	facebook.com	108
15	dailymail.co.uk	7661	summit.news	202	instagram.com	74
16	invidio.us	7639	iol.co.za	184	tgraph.io	74
17	dnaindia.com	6859	ewn.co.za	148	nalog.garant.ru	68
18	facebook.com	6641	express.co.uk	142	finance.yahoo.com	63
19	bitchute.com	6262	google.com	132	searchengines.ru	62
20	epochtimes.de	6243	m.news24.com	131	vc.ru	60

The top domains for in the main communities of the second network (July. 2019 to Feb. 2020) are displayed in Table 7. In the Russian downstream cluster of the second network, the top domain name is still rossaprimavera.ru as in the first network, but it is now accompanied by Russian news websites that were in the core Russian community before (e.g. lenta.ru, meduza.io, rt.com). A new domain, tasnimnews.com, is the website of a private news agency in Iran that publishes in Persian and English and is classified as a questionable source diffusing state propaganda by MBFC.^33^ The RBO value between the ranked lists of the first and second networks of this community is 0.36 indicating an important change of domain name usage (Table 9).

In the far-right downstream community, we see many of the same websites that were in the first network including popular center/left news outlets (e.g. nytimes.com, bbc.co.uk), US far-right news outlets (e.g. breitbart.com, dailycaller.com, zerohedge.com, infowars.com) and social media websites such as twitter.com, reddit.com, nitter.pro (an alternative Twitter front-end focused on privacy) and youtube.com. New appearances in the top 20 include the websites farsnews.ir from the Fars News Agency in Iran, known to be the “semi-official” news agency of the Government of Iran,^34^epochtimes.de, the German edition of the far-right international multi-language newspaper and media company affiliated with the Falun Gong new religious movement,^35^palinfo.com from the Palestinian Information Center, a Palestinian news website, timesofindia.indiatimes.com, an Indian daily newspaper with right-center bias and mixed factual reporting according to MBFC^36^ and fort-russ.com which is not active anymore but which has been flagged for disseminating Russian disinformation by EUvsDisinfo.^37^ For this community, the RBO value between the first and second network is 0.56 indicating again a less important change in domain name usage than in the Russian downstream community (Table 9).

The top domain names in the Russian core community of the second network are relatively similar (RBO of 0.53) to the ones in the same community of the first network with a mix of social media (YouTube, Twitter, Facebook, Instagram, VKontakte), Russian news outlets, many having been flagged as spreading pro-Russian disinformation by EUvsDisinfo (e.g. rbc.ru, govoritmoskva.ru, znak.com, kommersant.ru), others having not been flagged as such and reporting being independent (e.g. meduza.io, tvrain.ru), and western news outlets (telegraph.co.uk and forbes.ru).

In the far-right core community, the top domain names are also similar to the top domain names in the same community of the first network (RBO of 0.74). The most shared domains are the social media websites Twitter, YouTube, Reddit and 4chan. We also see the Russian propaganda outlets Sputnik News and RT, as well as far-right US and EU news outlets such as Info Wars, Zero Hedge, Voice of Europe and Epoch Times (in German). Domain names that appear for the first time include yna.kr, from the South Korean Yonhap News Agency, bitchute.com, an “alt-tech” video hosting service that plays the role of an alternative to YouTube for the far-right and where hate speech is highly prevalent (Trujillo et al. 2020; Freelon et al. 2020).

In the far-right upstream cluster, the social media YouTube, Twitter and Facebook are among the most shared domain names, as they were in the first network. The RBO value between the first and second networks is 0.60 (Table 9). BitChute is now also present in the list. Three South African news outlets also appeared on the list (businesstech.co.za, iol.co.za and ewn.co.za).

The top domain names in the Russian upstream community are mainly different than the ones in the same community for the first network (RBO of 0.14). The websites remaining in the top 20 include social media (YouTube, Twitter) and the blog hosting website Medium. Other top 20 websites include telegraph.co.uk and Russian news outlets.

**Table 8:** Top URL domains shared in the main communities of the network $G_3$ (March 2020 to Dec. 2020, Fig. 6)

	Downstream $D_F$		Downstream $D_R$		Core $C_F$	
	Domain	Count	Domain	Count	Domain	Count
1	twitter.com	131,267	rossaprimavera.ru	240,066	twitter.com	653,224
2	nytimes.com	31,059	lenta.ru	53,147	youtube.com	315,421
3	nournews.ir	30,833	tasnimnews.com	20,361	4chan.org	101,480
4	breitbart.com	28,538	tass.ru	10,039	imdb.com	28,015
5	4chan.org	28,342	meduza.io	6908	infowars.com	27,445
6	timesofindia.indiatimes.com	26,726	rt.com	5414	archive.is	24,931
7	dailycaller.com	26,004	mbk-news.appspot.com	3386	bitchute.com	20,265
8	readhacker.news	25,848	republic.ru	2615	facebook.com	17,707
9	cnn.com	21,651	warspot.ru	2283	voiceofeurope.com	17,418
10	news.ycombinator.com	20,217	telegraph.co.uk	1680	nitter.snopyta.org	16,677
11	zerohedge.com	15,815	instagram.com	1570	politaufkleber.de	16,030
12	bbc.co.uk	15,086	targetingsstorage.blob.core.windows.net	1110	instagram.com	10,736
13	youtube.com	14,656	gorky.media	1038	dlive.tv	9848
14	etherscan.io	13,853	youtube.com	770	telegraph.co.uk	9126
15	epochtimes.de	13,835	seance.ru	707	dailymail.co.uk	9036
16	uniswap.info	13,826	vk.com	693	breitbart.com	9011
17	farsnews.ir	13,060	topspb.tv	532	zerohedge.com	8979
18	ift.tt	11,615	discours.io	331	thegatewaypundit.com	8859
19	gate.io	9200	teletype.in	313	yts.mx	7765
20	dnaindia.com	9191	shop.seance.ru	193	travala.com	7368

	Core $C_R$		Upstream $U_F$		Upstream cluster (15)	
	Domain	Count	Domain	Count	Domain	Count
1	youtube.com	95,854	youtube.com	28,993	telegraph.co.uk	275
2	telegraph.co.uk	88,620	twitter.com	11,240	youtube.com	224
3	riafan.ru	26,062	breitbart.com	647	itzine.ru	109
4	instagram.com	25,714	facebook.com	631	twitter.com	88
5	vk.com	25,008	bitchute.com	602	seaforms.mod.mil.iq	70
6	facebook.com	23,035	reddit.com	584	instagram.com	66
7	rbc.ru	22,745	archive.is	510	vk.com	38
8	govoritmoskva.ru	22,732	thegatewaypundit.com	457	facebook.com	37
9	twitter.com	21,412	parler.com	446	iris-tg.ru	35
10	kommersant.ru	14,978	share.par.pw	438	gcpi-navy.com	34
11	kp.ru	14,592	infowars.com	402	drive.google.com	26
12	znak.com	14,568	summit.news	335	forms.gle	25
13	tass.ru	12,891	zerohedge.com	334	music.yandex.ru	23
14	meduza.io	12,125	dailymail.co.uk	331	podcasts.google.com	21
15	tvrain.ru	11,963	jaydafransen.online	316	apple.co	21
16	forbes.ru	11,319	ift.tt	305	mohesr.gov.iq	17
17	ria.ru	10,109	britishfreedomparty.com	297	dirasat-gate.org	14
18	novayagazeta.ru	9787	gorightnews.com	279	docs.google.com	12
19	rtvi.com	9782	newswars.com	274	pe-gate.org	11
20	news.tut.by	7643	en.m.wikipedia.org	272	teletype.in	10

**Table 9:** Rank-biased overlap similarity (RBO) (Webber et al. 2010) between the ranked domain lists of the first and second networks (first row), and second and third networks (second row) of the downstream, core and upstream Russian and far-right communities

	Downsteam		Core		Upstream	
	Russian	Far-right	Russian	Far-right	Russian	Far-right
$G_1$ to $G_2$	0.36	0.56	0.53	0.74	0.14	0.60
$G_2$ to $G_3$	0.78	0.71	0.67	0.64	0.38	0.65

We compute the RBO with a parameter $p=0.91$ such that the top 20 domains have 95% of the measure’s weight. The RBO value is between 0 and 1 with higher values indicating more similar ranked lists

In the third network (from March 2020 to Dec. 2020, Table 8), the two top domains in the far-right downstream community are still twitter.com and nytimes.com, but twitter.com has now more than four times more counts than nytimes.com. The rest of the top domain names are very similar to the domains list from the second network with an RBO value of 0.71 (Table 9). The Russian downstream and the Russian and far-right core communities also have very similar top domains than in the second network with RBO values of 0.78, 0.67 and 0.64, respectively.

The top domains in the far-right upstream community are also very similar to the domains in the second network (RBO of 0.65). We see the appearance of the new domain names parler.com, the American alt-tech social media service associated with Donald Trump supporters and far-right extremists,^38^ and britishfreedomparty.com which is a political party associated with the British far-right activist Jayda Fransen.^39^

## Discussion

We applied a community detection method that cluster flows of random walkers on the networks. Our community detection analysis reveals the existence of communities of channels with different roles. We find that the far-right sub-network in our Telegram dataset can be described by an upstream, a core and a downstream community. We also find a parallel sub-network mainly comprised of channels in the Russian language with a similar structure.

The upstream far-right community contains between 44 and 78 channels whose most important ones are mainly group chats, where users can all post messages and have discussions. Upstream communities are defined as a group of nodes from which the flow of random walkers tends to stay together and move to similar other channels, i.e. by following the link emanating from these channels, one ends up in the same channels, different than the starting group of channels. As the network evolves, only a small fraction of these channels remain in the upstream community, the majority moving to the core communities. The main links to external websites shared in the far-right upstream community point to social media (Twitter and Youtube are the most shared), indicating that content shared on these more traditional platforms is greatly discussed in the upstream group chats. The upstream community can be seen as being a place for users to discuss the content shared on external websites as well as the the content shared on broadcast channels in the core and the downstream communities. It can be seen as an entry point for users to learn about and access channels in these other part of the network.

The core far-right community grew considerably in size during our collection period (from 319 to 1252 channels). Core communities are defined by groups of channels from which the flow of random walker tend to stay inside the group, implying that they have reciprocal interactions and form a tight cluster. The most important channels in this community are mainly broadcast channels, i.e. channels where a small selected number of users (administrators) make posts that are read by the members of the channel. We find channels linked to far-right organizations and personalities in the UK and US as well as channels related to the anonymous message board 4Chan. Several channels in this community have been observed in other works investigating the far-right movement on Telegram (Urman and Katz 2022; Walther and McCoy 2021). As the network evolves, a large majority of channels remain in the core community and only a small fraction exit the system. The most shared domains in these communities are relatively stable across time and consist of social media websites (e.g. Twitter, YouTube, Reddit and 4chan) as well as Russian propaganda outlets (e.g. Sputnik News and RT) and far-right US and EU news outlets such as Info Wars, Zero Hedge, Voice of Europe and Epoch Times (in German). This community can be seen as made of a tight group of central channels that strongly interact with each other, share and comment on content from social media platforms and far-right news outlets.

The downstream far-right community has a stable size of around 2500 channels in the three networks corresponding to the different time periods. Downstream communities are defined as the opposite of upstream communities. When following the link starting from a downstream community in the reversed direction, one ends up on a similar group of channels that is different from the starting group, i.e. downstream communities are the ones linked by other channels in a non-reciprocal way. The most shared domain names in this community are well-known US and UK center and left-leaning traditional news outlets and US right-wing and far-right news outlets as well as news outlets having been flagged for propagating pro-Russian propaganda. Social media websites are also present in the top domain names. The channels in the downstream far-right community can be seen as representing the source of information and object of discussions of the far-right core and upstream channels.

Our investigation also revealed the presence of a Russian sub-network also organized in a main upstream, core and downstream community, with channels from Russian news outlets, most of them having been identified as spreading pro-Russian propaganda, and channels from Russian political figures and independent commentators. In the most shared domain name, we find many news outlets flagged by EUvsDisinfo for sharing disinformation but also some news outlets diffusing independent reporting about Russia (e.g. meduza.io). This suggests that discussions between pro-Russian government channels and channels critical of the government happen on Telegram. Investigating the extent and the evolution of such disputes on Telegram would be interesting. Moreover, investigating the relations between the Russian and US/UK far-right sub-networks would also be interesting. We have seen that pro-Russian news outlets in English were present in the most shared domains in the far-right sub-network.

The far-right core community we discovered can be seen as an “echo chamber”, i.e. an environment in which the opinion, political leaning, or belief of users about a topic gets reinforced due to repeated circular or reciprocated interactions with peers or sources having similar tendencies and attitudes (Cinelli et al. 2021). However, echo chambers take different forms depending on the medium in which interactions happen (Jamieson and Cappella 2008; Garrett 2009; Garimella et al. 2018; Cota et al. 2019; Cinelli et al. 2021). For a user navigating the network following links from channel to channel, the core communities, as we defined them, play indeed the role of echo chambers, however, we note that our network is defined at the level of channels and group chats and that the main far-right core community is mainly made of broadcast channels. In broadcast channels, users are mostly consuming the content and not actively participating in discussions. On the other hand, in group chats, users can discuss with each other and it is likely that opinion-reinforcing processes also take place in group chats. Interestingly, we found many group chats in the far-right upstream community, indicating that these group chats link more frequently to the core community than between themselves. This suggests two different levels of echo chambers: a participative one inside group chats and a more passive one made of the channels of the core community. In our case, an interesting line of research would also be to better understand the structure of the echo chamber in the core community. Indeed, the core community is made of a large number of channels that may focus on different topics and have different objectives. When investigating the far-right network on Telegram, Urman and Katz found several communities divided mainly along ideological and national lines (Urman and Katz 2022). A possibility would be to define finer echo chambers as graph cycles, i.e. directed paths across channels in which all edges are distinct and that start and end on the same channel. They would capture the reinforcing effect of echo chambers. One could then decompose the core community into individual cycles and characterize their overlap, possible independence, and evolution.

Our approach allowed us to provide a novel insight onto the organization of the far-right network in Telegram revealing the different roles that have different types of channels. Our method could be applied to uncover the organization of interactions between different actors in other social media platforms where interactions take place between user groups (e.g. subreddits, Facebook groups) or directly between users (e.g. Twitter). Further work could focus on better characterizing the echo chambers in the core community and also better understanding the limitations inherent to the method of collection. For example, following links posted on a seed set of channels could explain why we find more channels in the downstream communities than in the upstream communities. Another aspect of Telegram data collection is the fact that users often delete their messages and that channel can also be deleted. Modeling these processes to better understand their effect on the inferred structure of the network would be an interesting line of research.

## Data Availability

The raw Telegram dataset collected during the current study is not publicly available as it could compromise individual privacy but it is available from the corresponding author on reasonable request. Complete edge lists of the three networks, where channel names have been replaced by their identification numbers, have been deposited in the Harvard Dataverse Repository at the following address https://doi.org/10.7910/DVN/7JUYII. Python codes allowing to apply the flow stability method to this dataset and to reproduce the clustering is also available at the same address. The flow stability package is available at https://github.com/alexbovet/flow_stability.

## Footnotes

1 https://telegram.org/blog/400-million.

2 https://www.europol.europa.eu/newsroom/news/europol-and-telegram-take-terrorist-propaganda-online.

3 https://en.wikipedia.org/wiki/Britain_First.

4 https://en.wikipedia.org/wiki/Far-right_politics_in_the_United_Kingdom.

5 https://mediabiasfactcheck.com/breaking911/.

6 https://en.wikipedia.org/wiki/Olavo_de_Carvalho.

7 https://en.wikipedia.org/wiki/Red_Color.

8 https://en.wikipedia.org/wiki/RT_(TV_network).

9 https://en.wikipedia.org/wiki/Maxim_Kononenko.

10 https://en.wikipedia.org/wiki/National_Bolshevism.

11 https://www.independent.co.uk/tech/telegram-white-supremacists-violence-black-lives-matter-blm-a9586911.html.

12 https://en.wikipedia.org/wiki/Milo_Yiannopoulos.

13 https://en.wikipedia.org/wiki/Tommy_Robinson_(activist).

14 https://en.wikipedia.org/wiki/Paul_Joseph_Watson.

15 https://en.wikipedia.org/wiki/Laura_Loomer.

16 https://mediabiasfactcheck.com/voice-of-europe/.

17 https://twitter.com/ErnestMakarenko.

18 https://mediabiasfactcheck.com/breitbart/.

19 https://www.tagesspiegel.de/themen/reportage/nach-streit-um-maskenpflicht-rechte-jubeln-ueber-mord-von-idar-oberstein/27631262.html.

20 https://en.wikipedia.org/wiki/Margarita_Simonyan.

21 https://en.wikipedia.org/wiki/Sidney_Powell.

22 https://www.bbc.com/news/uk-northern-ireland-45562784.

23 https://www.bbc.co.uk/news/election-us-2020-54359993.

24 https://twitter.com/newsfromdonbass.

25 https://euvsdisinfo.eu/disinformation-cases/.

26 https://mediabiasfactcheck.com/south-front.

27 https://mediabiasfactcheck.com/russia-insider.

28 https://en.wikipedia.org/wiki/Meduza.

29 https://en.wikipedia.org/wiki/NTV_(Russia).

30 https://euvsdisinfo.eu/disinformation-cases/.

31 https://mediabiasfactcheck.com/zero-hedge/.

32 https://en.wikipedia.org/wiki/The_Daily_Stormer.

33 https://mediabiasfactcheck.com/tasnim-news-agency/.

34 https://en.wikipedia.org/wiki/Fars_News_Agency.

35 https://en.wikipedia.org/wiki/The_Epoch_Times.

36 https://mediabiasfactcheck.com/times-of-india/.

37 https://euvsdisinfo.eu/disinformation-cases/?text=fort-russ.com.

38 https://en.wikipedia.org/wiki/Parler.

39 https://en.wikipedia.org/wiki/Jayda_Fransen.
